# Changes in Energy Expenditure Determined by Indirect Calorimetry in Severe Burn Patients During the Acute Phase

**DOI:** 10.7759/cureus.46705

**Published:** 2023-10-09

**Authors:** Tuba Kuvvet Yoldaş, Alev Atalay, Kubilay Demirağ, Mehmet Uyar, İlkin Çankayalı

**Affiliations:** 1 Clinic of Anesthesiology and Reanimation, University of Health Sciences, Tepecik Education and Research Hospital, İzmir, TUR; 2 Department of Anesthesiology and Reanimation, Faculty of Medicine, Ege University, İzmir, TUR

**Keywords:** severe burn patient, basal metabolic rate, adult burn patients, indirect calorimetry, resting energy expenditure

## Abstract

Background: Severe burn injuries are a major health problem globally. A profound and prolonged hypermetabolic response develops in severe burn injuries and it is crucial to monitor the patients’ energy requirements in order to meet them adequately. The aim of the present study was to examine the energy changes during the acute phase using the indirect calorimetry (IC) method in severe burn patients.

Methods: The study included 15 severe burn patients. Patients with FiO_2 _>60%, tube thoracostomy, closed underwater drain (CUWD) and air leakage were excluded from the study. Patients’ demographic data, burn percentages, burn types, duration of stay in intensive care, mortality and Acute Physiology and Chronic Health Evaluation II (APACHE II) scores were recorded. Indirect calorimeter measurements were taken once from the patients upon their first arrival and during the following four weeks. Resting energy expenditure (REE), basal metabolic rate (BMR), oxygen consumption (VO₂), carbon dioxide production (VCO₂), body temperatures, presence of sepsis, Sequential Organ Failure Assessment (SOFA) and Modified Nutrition Risk in Critically Ill (mNUTRIC) scores were recorded. The data were analysed using SPSS 24 and p-values <0.05 were considered statistically significant.

Results: In the study, 13 (86.67%) of the patients were male. Patients' mean age was 45.27±18.16 years, and mean BMI 25.99±4.22 kg/m^2^. Five patients (33.33%) had chronic diseases. The average burn percentage was 45%, with 7 (46.67%) patients having a burn percentage of ≤40%, while 8 (53.33%) had a burn percentage of >40%. A total of 14 (93.33%) had flame burns; 3 (20.00%) patients deceased, and 12 (80.00%) were discharged. The mean APACHE II score was 11.53±6.83. The measured mean values of REE, VO₂, VCO₂ and fever were seen to be the highest in the first week after admission and decreases were observed in the subsequent weeks. SOFA score averages were the highest at admission, and decreased in the following weeks.

Conclusion: Severe burn patients were observed to go through the hypermetabolic process in the acute phase and their energy requirements were high particularly in the first week. It was concluded that regular IC monitoring can be beneficial to fully meet the energy requirements of severe burn patients due to the prolonged hypermetabolic process.

## Introduction

A severe and prolonged hypermetabolic response develops in severe burn injuries, which are among significant health problems. This hypermetabolic response depends on the endocrine stress response and inflammatory response (multiple mediators) as well as the classical factors of age, gender, wound size and healing time. There is an increased energy requirement due to the prolonged hypermetabolic process [1]. Energy requirement in burns is caused by the significant increase in resting energy expenditure (REE). This increase is time-variable and proportional to the burned body surface area. REE represents approximately 70% of the total energy expenditure. The metabolic rate can surpass twice the basal rate and this response can last for more than one year following the injury [2]. It is necessary to determine the energy requirement for nutritional support in these patients. Accurate identification of nutritional needs is crucial and malnutrition must be prevented because it is associated with adverse outcomes such as nosocomial infections, prolonged hospital stays, failure to wean off mechanical ventilation and mortality. On the other hand, overnutrition can lead to unfavourable complications such as increased fat storage, hyperglycaemia and elevated CO₂ [3,4]. Due to the harmful outcomes of inadequate and excessive nutrition, the patient’s energy requirement must be determined precisely. Optimal nutrition is an important issue for care; burn-related metabolic changes must be well understood and the actual energy expenditure of patients must be determined accurately. Indirect calorimetry (IC) is currently recommended as the most accurate method for evaluating measured values, but it is still not widely used in clinical practice [5]. There are few studies present in the literature using IC in deep burn patients [6].

Indirect calorimetry is the gold standard in determining the total energy requirement and calculates the energy consumption based on an individual’s oxygen consumption (VO₂) and carbon dioxide production (VCO₂) using the Weir equation [7]. Regular use of IC ensures the prevention of insufficient/excessive nutrition through adequate nutritional support in patients with severe burns.

The aim of our study was to examine the energy changes in the acute phase using the indirect calorimetry method in severe burn patients in the intensive care unit and to determine the factors affecting energy changes.

## Materials and methods

Out of the 20 patients admitted between April and December, 2019, in the burn intensive care unit, 15 patients were included in the study. Five patients were excluded since their duration of stay in the intensive care was shorter than 48 hours. The study was approved by the Clinical Research Ethics Committee, Ege University Faculty of Medicine (approval no. 19-5.2T/8). Patients with FiO_2 _>60%, tube thoracostomy, closed underwater drain (CUWD) and air leakage were excluded from the study. Patients’ demographic data, burn percentages, burn types, duration of stay in intensive care, mortality and Acute Physiology and Chronic Health Evaluation II (APACHE II) scores were recorded. Indirect calorimeter measurements were taken once from the patients upon their first arrival and during the following four weeks. Resting energy expenditure, basal metabolic rate (BMR), oxygen consumption (VO₂), carbon dioxide production (VCO₂), body temperatures, presence of sepsis, Sequential Organ Failure Assessment (SOFA) and Modified Nutrition Risk in Critically Ill (mNUTRIC) scores were recorded. Patients’ daily energy requirements were determined using a metabolic monitor (Quark REE indirect calorimeter; COSMED, Italy) with the IC method. Indirect calorimeter measurements were conducted using three different methods. Canopy hoods or face masks were used for patients who were non-intubated and spontaneously breathing. For intubated/tracheostomized patients, measurements were conducted using a device attached to the endotracheal tube. The energy requirements of the patients were measured using an indirect calorimeter every week, on the same day, in the early morning hours, and for a duration of four weeks. Patients’ nutritional treatments were revised on the basis of their results.

In the present study, we evaluated the changes in the energy requirements of severe burn patients based on measurements taken over a four-week period. The predictive factors influencing energy changes were examined.

Data collected from the study were analyzed using IBM® SPSS Statistics, version 24 (IBM Corp., Armonk, NY). Descriptive statistics regarding the distribution of the responses to the independent variables are presented as numbers and percentages for categorical variables, and means, standard deviations and median for numeric variables. For paired and multiple comparisons, the chi-square test was performed for categorical variables and independent t-test and one-way ANOVA test for quantitative variables. The Tukey test was applied for the comparison among more than two groups of quantitative variables. The results were considered statistically significant at a confidence interval of 95% with p<0.05.

## Results

Of the 15 patients, 13 (86.67%) were male, and 2 (13.33%) were female. The average age of the patients was 45.27±18.16 years. The mean BMI was calculated as 25.99±4.22 kg/m². The mean APACHE II score was 11.53±6.83. The average length of stay for patients in the intensive care unit was 50.93±29.36 days. Five (33.33%) patients had comorbidities. Patients’ mean total body surface area (TBSA) of burn was 45%; 7 (46.67%) patients had TBSA ≤40%, while 8 (53.33%) had TBSA >40%. In addition, 3 patients had first- and second-degree burns (20%) and 12 patients had second- and third-degree burns (80%). As for the burn etiology, the most frequent cause was flame burns (93.33%). Only one of the patients had an inhalation burn. Three patients (20%) were monitored with the support of mechanical ventilation and inotropes. Three patients (20.00%) died, while 12 (80.00%) were discharged (Table 1).

**Table 1:** Demographic data of patients Data is represented as n (%). BMI: body mass index, TBSA: total body surface area, APACHE II: Acute Physiology and Chronic Health Evaluation II, SOFA: Sequential Organ Failure Assessment, mNUTRIC: Modified Nutrition Risk in Critically Ill

Demographics				
Gender, n (%)	Female			2 (13.33%)
	Male			13 (86.67%)
Age, mean±SD				45.27±18.16
Height (cm), mean±SD				173.73±8.15
Weight (kg), mean±SD				78.13±11.56
BMI (kg/m^2^), mean±SD				25.99±4.22
Chronic disease, n (%)		Yes		5 (33.33%)
		No		10 (66.67%)
Duration of intensive care stay (days)				50.93±29.36
APACHE II score, mean±SD				11.53±6.83
SOFA score (admission), mean±SD				2.6±2
mNUTRIC score (admission), mean±SD				1.74±1.79
TBSA, mean (%)				45%
TBSA, n (%)			≤40	7 (46.67%)
			˃40	8 (53.33%)
Degree of burn, n (%)			1st-2nd	3 (20%)
			2nd-3rd	12 (80%)
Mortality, n (%)			Yes	3 (20%)
			No	12 (80%)

The mean values measured at the admission of the patients were REE 2474.73±386.06 kcal/day and BMR 1780.00±373.35 kcal/day (using Harris-Benedict formula). In the subsequent measurements, mean REE, BMR, VO₂, VCO₂ and fever values were observed to the highest in the first week, while they tended to decrease in the following weeks. The mean SOFA and mNUTRIC scores had the highest values on the first day whereas decreases were observed in the following weeks (Table 2). All the REE measurements were found to be higher than the BMR measurements.

**Table 2:** Mean REE, BMR, VO2, VCO2, fever, SOFA score and mNUTRIC score changes in patients (at admission and in the first four weeks) Data is represented as means±SDs. Statistically significant changes were observed in the values measured in the first and second weeks compared to the values measured at admission. *Results were considered statistically significant at p<0.05. BMR: basal metabolic rate, REE: resting energy expenditure, VO_2_: oxygen consumption, VCO_2_: carbon dioxide production, SOFA: Sequential Organ Failure Assessment, mNUTRIC: Modified Nutrition Risk in Critically Ill

	BMR (kcal/day), mean±SD	REE (kcal/day), mean±SD	VO_2_ (L/min), mean±SD	VCO_2_ (L/min), mean±SD	Body temperature (˚C), mean±SD	SOFA, mean±SD	mNUTRIC score, mean±SD
Admission	1780±373.3	2474.7±386	364.6±62.6	256.2±52.1	37.4±0.8	2.6±2	1.74±1.79
Week 1	1787.2±374.3	2837.9±634.2*	416.6±91.9*	314.7±84.1*	37.9±0.6	2.6±2.2	1.73±1.83
Week 2	1779±393.9	2689.14±462.8*	394.8±72.2*	297.8±48.1*	37.7±0.5	2.07±2.8	1.5±1.79
Week 3	1780±395.2	2460.7±428.9	358.7±68.4	272.07±51.3	37.2±0.5	1.9±2.7	1.36±1.65
Week 4	1785±440.7	2512.4±501.4	366.7±76.3	286.5±52.2	36.9±0.5	1.9±0.8	1.3±0.6

Changes in the REE compared to the BMR at admission and over the subsequent four weeks were measured with indirect calorimetry and found to increase by 38.9%, 58.76%, 51.15%, 38.20% and 40.73%, respectively (Table 3).

**Table 3:** BMR and REE changes (%) by weeks, at admission and in the first four weeks Data is represented as means±SDs and percentages. BMR: basal metabolic rate, REE: resting energy expenditure

	BMR (kcal/day), mean±SD	REE (kcal/day), mean±SD	Change (%)
Admission	1780±373.3	2474.7±386	38.99 ↑
Week 1	1787.2±374.3	2837.9±634.2*	58.76 ↑
Week 2	1779±393.9	2689.14±462.8*	51.15 ↑
Week 3	1780±395.2	2460.7±428.9	38.20 ↑
Week 4	1785±440.7	2512.4±501.4	40.73 ↑

In our study, REE was found to be significantly higher among the male patients compared to the female patients (p=0.016); patients' ages ranged between 18 and 80 years. REE was observed to be higher and statistically significant particularly in patients aged 30-50 (p=0.048). Higher BMI values were associated with elevated REE values. In patients with BMI ≥24.3, a statistically significant increase was found in the REE (p=0.034). Energy requirements were observed to be higher in patients whose TBSA was above 37%. It was found that TBSA ≤40% or >40% did not create a significant difference in REE. REE values were statistically higher in patients who required inotropic support and mechanical ventilation (p=0.036). In the 11 patients with sepsis, REE values were found to be high at a statistically significant level (p=0.038). In patients with elevated SOFA and mNUTRIC scores, REE values were statistically significantly high (p=0.036, p=0.009). However, no significant difference was found between the APACHE score and high REE values (p=0.571). As the length of stay in the intensive care prolonged, statistically significant increases were observed in REE values (p=0.011) (Table 4). It was observed that REE values were significantly high in patients who died (p=0.036).

**Table 4:** Factors affecting REE increase Data is represented as means±SDs. *Results were considered statistically significant at p<0.05. BMI: body mass index, TBSA: total body surface area, REE: resting energy expenditure, SOFA: Sequential Organ Failure Assessment, mNUTRIC: Modified Nutrition Risk in Critically Ill

		REE (kcal/day), mean±SD	p-value
Gender*	Female	1884.5±324.6	0.016
	Male	2984.6±534.5	
	18-30	2338±515.2	
Age (years)*	30-50	3311.8±520.8	0.048
	˃50	2486.5±489.5	
BMI (kg/m²)*	18-25	2494.6±373.3	0.034
	25-30	3186.3±123.4	
	30-35	2497±527.2	
TBSA (%)	≤40%	2668±661.1	0.35
	>40%	2986.6±612.9	
Endotracheal mechanical ventilation + inotrope/vasopressor support*	Yes	2974.5±589.9	0.036
	No	2291.7±589	
Sepsis*	Yes	3134.8±690.4	0.038
	No	2730±610	
SOFA score*	˂2	2709.1±482.05	0.036
	≥2	2943.7±634.2	
mNUTRIC score*	˂3	2458.1±523.4	0.009
	≥3	2942.3±432.7	
Mortality*	Exitus	2974.5±589.9	0.036
	Discharged	2291.7±589	

## Discussion

Determining daily energy and protein requirements accurately is a critical part of managing the nutrition treatment in burn patients. Accelerating the healing of the wound, shortening the hospital stay by preventing additional complications and reducing mortality/morbidity through comprehensive nutrition treatment planning are among the main goals in the management of the burn intensive care patient [8].

Hypermetabolic response changes over time in severe burns [9]. Energy need increases in the 72 hours following the burn trauma, reaching its peak points on the fifth and seventh days [10]. There are studies reporting that metabolic increases occur in the first two weeks. In the study conducted by Guo and colleagues, the first IC measurements after burn trauma were taken in the first week and the patients were monitored for 60 days. The highest increase in the energy requirement of the patients was observed to occur in the first week and gradual decreases were recorded in the measurements taken in the following weeks. The first three weeks were reported as the period when the energy requirement was the highest [11]. Consistent with the literature, in our study, mean REE values measured were observed to increase as of the first week. The REE value measured after the third week was seen to begin decreasing compared to the initial REE value.

In the study carried out by Phan et al., initial REE and BMR measurements of 62 severe burn patients were taken with IC on the third day and the measurements were continued during the subsequent four weeks. Specifically on the seventh day, REE values were found to be much higher than the BMR values. The difference between REE and BMR was observed to be high throughout the four weeks [12]. The study conducted by Guo and colleagues, on the other hand, reported that REE was far higher than the BMR for four weeks, stating that the hypermetabolic process extended as long as this period [11]. In our study, although declines were observed in REE after one week, the hypermetabolic process was seen to continue for four weeks compared to the BMR. Changes in REE compared to the BMR were measured at admission and during the following four weeks and were found to increase by 38.9%, 58.76%, 51.15%, 38.20% and 40.73%, respectively. Phan et al. reported the REE changes compared to BMR on days 3, 7, 14, 21 and 28 as 61.2%, 48.2%, 49%, 52.4%, 49.9%, respectively [12].

IC is considered the gold standard in calculating daily energy requirements [1]. Taking IC measurements is crucial, especially in burn intensive care patients, where the course is hypermetabolic, catabolism takes a long time and energy requirements may vary. Weekly measurements taken in the present study revealed that energy requirements change from week to week (Table 2 and Table 3). The factors that can affect energy consumption following burn include the size and depth of the burn, surgical procedures, timing of initiating nutrition, physical therapy, medications, sepsis and ambient temperature. Factors such as age, gender and BMI are also influential.

While examining the effect of age on REE, it was found to have no effect in the study conducted by Phan and colleagues that included adult severe burn patients [12]. However, in the study carried out by Kim et al. in 2009, of the 199 adult patients with ≥20% TBSA, those aged 18-40 were found to have higher REE values [13]. Similarly, energy requirement was observed to be higher in the age group of 30-50 years in our study (p=0.048).

Younger age groups have greater energy consumptions due to their physical activity [14]. Particularly, young age and male gender are strong factors affecting REE. Phan et al. found that male patients had significantly higher levels of REE compared to female patients on the 3rd, 7th and 21st day after the burn [12]. Guo and colleagues reported that male patients with the same burn type had higher REE values [11]. Similarly in our study, male patients’ mean REE values were significantly higher than those of the female patients, indicating a significant difference (p=0.016).

In many studies, TBSA is addressed as one of the factors affecting REE. In the study conducted by Stanojcic and colleagues, when patients with a burn TBSA of 20%-40% and those with a >40% TBSA were examined, energy requirements were observed to be similar in the two groups [15]. On the other hand, Phan and colleagues found that REE was significantly higher in patients with second- and third-degree burns together with TBSA ≥60% or TBSA ≥20% [12]. In our study, 80% of the patients had second- to third-degree burns. It was found that TBSA ≤40% or TBSA >40% had no significant effect on REE.

There are different opinions regarding the effect of surgical intervention on the metabolic response. According to Williams et al. and Hart et al., there is a significant decrease in the metabolic response with early excision (<72 hours after the burn) compared to delayed excision (one week after the burn) [16,17]. However, in another study conducted by Hart et al., no difference could be found between early and delayed excision [18]. Since all our patients were operated within the first week following the burn trauma, we were not able to evaluate the effect of operating time on energy requirements.

The mortality rate was observed as 20% in our study. In the study carried out by Phan et al., the mortality rate was reported as 17.7%. The literature states that mortality and REE are correlated [12]. Jeschke et al. examined 230 paediatric patients who had burns exceeding TBSA 30% and underwent at least one surgical operation, and found that there was a higher hypermetabolic response in non-survivors compared to survivors due to increased organ dysfunction and sepsis [19,20]. Similarly, in our study, REE values were measured to be higher among non-survivors.

Inhalation injury is also one of the factors that increase mortality in burn patients. Our only patient with inhalation injury had been a smoker for many years. There are many fatal diseases associated with smoking [21]. We are curious about whether our patient's inhalation injury and heavy smoking accelerated mortality; this association can be further investigated.

Limitations of this study included a small sample size. Some of our patients received treatment within 48 hours and were subsequently discharged from intensive care. They were excluded from the study because it was not possible to obtain sufficient ICU measurements. The treatment duration for our other patients extended beyond four weeks, and the number of patients we were able to follow was limited. Furthermore, the population distribution being studied skewed toward middle-aged individuals. In our study, there was a higher number of patients in the young to middle-aged group, and hence, our data predominantly reflects that population.

## Conclusions

The results show that severe burn patients go through a hypermetabolic process in the acute period and their energy requirements are higher, especially in the first week. The hypermetabolic process lasts up to three weeks. Since the hypermetabolic process takes a long time, it is suggested that regular indirect calorimetry monitoring would be beneficial in order to adequately meet the energy needs of severe burn patients. Considering the factors that increase energy needs and making regular indirect calorimetry measurements would accelerate the recovery process of patients and shorten the intensive care stay.
